# Pancreatic Steatosis Evaluated by Automated Volumetric CT Fat Fraction of the Pancreas: Association with Severity in COVID-19 Pneumonia

**DOI:** 10.3390/tomography8060234

**Published:** 2022-11-24

**Authors:** Masahiro Tanabe, Yoshie Kunihiro, Mayumi Higashi, Kenichiro Ihara, Masaya Tanabe, Takeshi Yagi, Taiga Kobayashi, Takaaki Ueda, Katsuyoshi Ito

**Affiliations:** 1 Department of Radiology, Yamaguchi University Graduate School of Medicine, 1-1-1 Minami-Kogushi, Ube, Yamaguchi 755-8505, Japan; 2 Advanced Medical Emergency and Critical Care Center, Yamaguchi University Hospital, Ube, Yamaguchi 755-8505, Japan

**Keywords:** coronavirus disease 2019, pancreatic steatosis, lung severity score, CT fat volume fraction

## Abstract

This study investigated the relationship between the severity of pneumonia based on chest CT findings and that of pancreatic steatosis assessed using an automated volumetric measurement of the CT fat volume fraction (CT-FVF) of the pancreas, using unenhanced three-dimensional CT in polymerase chain reaction (PCR)-confirmed COVID-19 patients. The study population consisted of 128 patients with PCR-confirmed COVID-19 infection who underwent CT examinations. The CT-FVF of the pancreas was calculated using a histogram analysis for the isolation of fat-containing voxels in the pancreas. The CT-FVF (%) of the pancreas had a significantly positive correlation with the lung severity score on CT (*ρ* = 0.549, *p* < 0.01). CT-FVF (%) of the pancreas in the severe pneumonia group was significantly higher than that of the non-severe pneumonia group (21.7% vs. 7.8%, *p* < 0.01). The area under the curve of CT-FVF (%) of the pancreas in predicting the severity of pneumonia on CT was calculated to be 0.82, with a sensitivity of 88% and a specificity of 68% at a threshold for the severity score of 12.3. The automated volumetric measurement of the CT-FVF of the pancreas using unenhanced CT can help estimate disease severity in patients with COVID-19 pneumonia based on chest CT findings.

## 1. Introduction

Coronavirus disease 2019 (COVID-19) caused by severe acute respiratory syndrome coronavirus 2 (SARS-CoV-2) can manifest as an asymptomatic disease in mild cases, and can progress to pneumonia, acute respiratory distress syndrome, and multi-organ dysfunction in severe cases [1,2]. Among patients diagnosed with COVID-19 infection, it has been reported that comorbidities, such as hypertension (HT), chronic obstructive pulmonary disease (COPD), chronic kidney disease, and cardiovascular disease, as well as diabetes mellitus (DM) and obesity, tend to increase the risk of severe disease [3]. The relationship between fatty liver and COVID-19 has also been investigated in a limited number of studies, and pre-existing fatty liver was associated with a severe course of COVID-19 [3,4].

Chest computed tomography (CT) has played an important role in the diagnosis as well as determination of the severity and prognosis of COVID-19 disease [5], and previous studies have shown that patients with a fatty liver had higher pneumonia severity scores and higher hospitalization rates when infected with COVID-19 than those without a fatty liver [4,6]. Thus, identifying patients with pre-existing fatty liver disease is important in the early stage of the disease [7].

Pancreatic steatosis has also been associated with obesity and DM [8], but its relationship with the severity of COVID-19 infection has not been fully investigated [9]. Several studies have assessed the presence of pancreatic steatosis on unenhanced CT using region of interest (ROI) measurements on the pancreas in selected images [10,11]. However, these measurements are not necessarily representative in cases with heterogeneous pancreatic fatty deposition. Recently, an automated volumetric measurement technique of the pancreas using three-dimensional (3D) CT data was developed, allowing the quantification of the fat volume within the pancreatic parenchyma (CT fat fraction of the pancreas). It would be useful to assess the risk of severity of COVID-19 based on the degree of pancreatic steatosis on unenhanced CT. The present study therefore investigated the relationship between the severity of pneumonia based on chest CT findings and the presence of pancreatic steatosis assessed using the automated volumetric measurement of the CT fat fraction of the pancreas, using unenhanced 3D-CT in polymerase chain reaction (PCR)-confirmed COVID-19 patients.

## 2. Materials and Methods

### 2.1. Study Population

**Figure 1:**
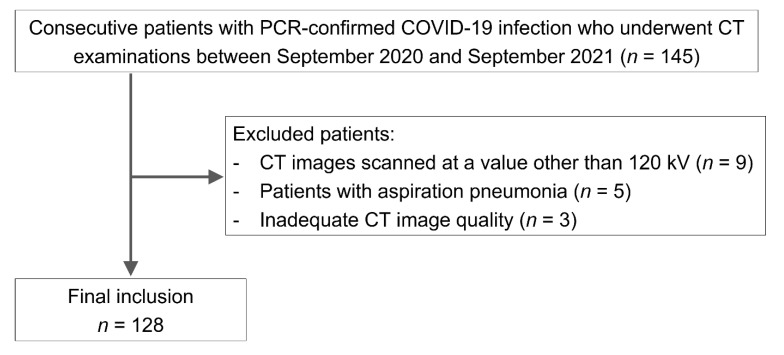
Flowchart of patient selection.

This retrospective study received institutional review board approval, and the requirement for written informed consent was waived. Consecutive patients with PCR-confirmed COVID-19 infection who underwent CT examinations between September 2020 and September 2021 were identified using our hospital information system. Of these patients, 17 were excluded for the following reasons: (1) CT images scanned at a value other than 120 kV (*n* = 9), (2) patients with aspiration pneumonia (*n* = 5), and (3) an inadequate CT image quality (e.g., presence of severe motion artifacts preventing the adequate assessment of CT findings) (*n* = 3). The flowchart for patient selection is shown in Figure 1. Ultimately, 128 patients (male, *n* = 80; female, *n* = 48; mean age, 55.4 ± 20.6 [range, 14–97] years old; nine teenagers, seven in their 20s, ten in their 30s, twenty-six in their 40s, twenty-four in their 50s, seventeen in their 60s, seventeen in their 70s, fourteen in their 80s, and four in their 90s) were included in the present study.

### 2.2. CT

CT was performed on a multidetector CT (MDCT) scanner (Optima CT660 Pro; GE Healthcare, Milwaukee, WI, USA; SOMATOM Drive; SIEMENS Healthineers, Erlangen, Germany) at 180–250 mA and 120 kVp. Unenhanced CT scans were obtained for all patients in a supine position and during a single deep inspiratory breath hold for the data analysis. The imaging parameters were as follows: detector collimation, 64 × 0.625 or 128 × 0.6 mm; pitch, 1.531 or 1.2; gantry rotation speed, 0.5 s; matrix, 512 × 512; section thickness, 1–1.25 mm; reconstruction interval, 1 mm. The tube voltage was set to 120 kVp. The tube current was determined by the automatic exposure control system.

### 2.3. Image Analyses

The automated volumetric measurement of the CT fat fraction of the pancreas on unenhanced CT was performed using a dedicated post-processing tool (SYNAPSE VINCENT; Fujifilm Corporation, Tokyo, Japan) on the workstation by 1 radiologist (M.T., 20 years of experience in body imaging), without access to chest CT findings or any clinical information except for patients’ PCR positivity.

In the first step, the pancreatic volume was measured in cm^3^ using all 1-mm slices of unenhanced CT images, using an automated segmentation program. With this program, the boundary of the pancreatic parenchyma was automatically outlined in all slices, and the pancreatic volume was calculated by the automatic summation of the segmented pancreatic area. The segmentation error of the pancreas was manually modified if the correction was necessary.

**Figure 2:**
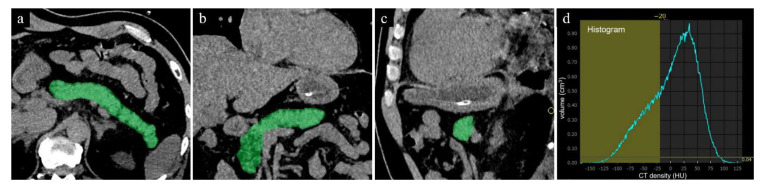
The measurement of the pancreatic fat volume on unenhanced CT. (**a**) axial, (**b**) coronal, (**c**) sagittal CT images. The pancreatic whole volume was measured in cm^3^ on unenhanced CT images using all slices, using an automated segmentation program installed in the workstation. (**d**) The pancreatic fat volume within the pancreas was calculated by means of a histogram analysis with local thresholding.

In the second step, the CT fat volume fraction (FVF) of the pancreas was calculated using a histogram analysis with thresholds of −190 to −20 HU for the isolation of fat-containing voxels in the pancreatic parenchyma (Figure 2). The threshold value of −20 HU was applied based on the preliminary results of our study performed to establish the best possible measure of fat content in the pancreas, although the CT value of fat was defined as <0 HU. Thus, the CT FVF (%) of the pancreas was defined as the volumetric percentage of pancreatic fat to total pancreatic volume [12].

Chest CT findings were assessed by a board-certificated experienced thoracic radiologist with 21 years of experience (Y.K.) on a picture archiving and communication system (PACS) workstation (ShadeQuest/ViewR-DG; FUJIFILM Medical Solutions, Tokyo, Japan) to determine the severity of COVID-19 pneumonia, according to the approach taken in a previous study [1]. Final decisions were reached by consensus with another radiologist (M.T.). CT images were reviewed for the following findings: (a) presence of ground-glass opacities, (b) presence of consolidation, and (c) number of lobes affected by ground-glass or consolidative opacities. Each of the five lung lobes was assessed for the degree of involvement and classified as none (0%) = a lobe severity score of 0, minimal (1–25%) = a lobe severity score of 1, mild (26–50%) = a lobe severity score of 2, moderate (51–75%) = a lobe severity score of 3, or severe (76–100%) = a lobe severity score of 4. The overall lung severity score was determined by averaging the total lobe scores (total lobe scores/total lobe numbers, range of possible scores, 0–4.0). According to the previous study [13], a lung severity score of ≥2.1 was defined as severe pneumonia based on the clinical outcome, while a lung severity score of <2.1 was defined as non-severe pneumonia.

The measurement of the CT attenuation values of the liver was also performed on the same PACS workstation. ROIs (size, >300 mm^2^) were placed at 2 different locations (right and left lobe) of the liver parenchyma on unenhanced CT images, and CT attenuation values (HU) in the liver were measured by the same radiologist who measured the CT fat fraction of the pancreas. The accuracy was verified by a senior radiologist (K.I., 34 years of experience). The averaged values measured at the right and left lobe of the liver were used for the data analysis.

### 2.4. Statistical Analyses

The Shapiro—Wilk test was performed to assess whether data were normally distributed. Normally distributed continuous data were expressed as the mean and standard deviation, while non-normally-distributed data were expressed as the median and interquartile range (IQR). The relationships between the age, CT-FVF (%) of the pancreas and CT attenuation values (HU) of the liver and the overall CT lung severity score were assessed using Spearman’s rank correlation coefficient. The differences of severe pneumonia and non-severe pneumonia were statistically compared using the Mann–Whitney U test. *p* values of <0.05 were considered to indicate a statistically significant difference. The accuracy of the CT-FVF (%) of the pancreas and CT attenuation values (HU) of the liver for predicting the severity of pneumonia in CT was evaluated by constructing a receiver operating characteristics (ROC) curve and calculating the area under the curve (AUC). The optimal threshold was identified by calculating the Youden index [14] to determine the sensitivity and the specificity.

All statistical analyses were performed using the SPSS software program (version 27.0; IBM, Armonk, NY, USA), and the Youden index was determined using the JMP Pro software program (version 16; SAS Institute Inc., Cary, NC, USA).

## 3. Results

**Figure 3:**
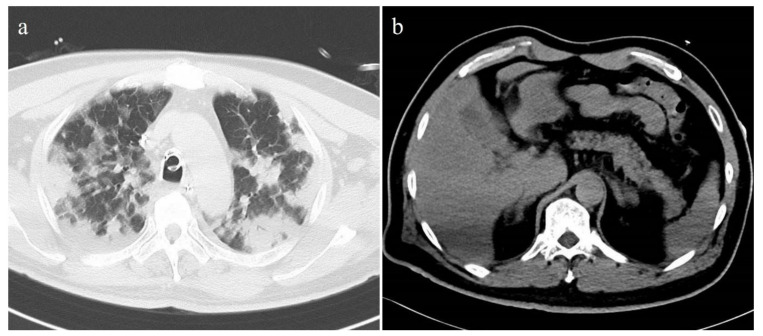
Unenhanced axial CT images of a 51-year-old male patient with severe COVID-19 pneumonia. (**a**) Chest CT showed extensive consolidations in both lungs. The lung severity score was 3.8. (**b**) Pancreatic steatosis and fatty liver were seen on the abdominal CT. The CT-FVF of the pancreas was 28.4% and the CT attenuation value of the liver was 46.9 HU.

**Figure 4:**
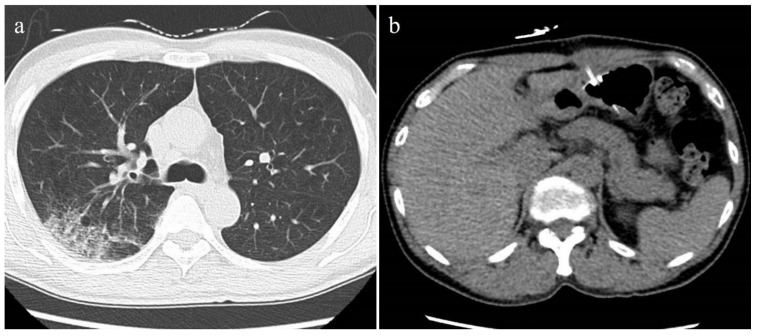
Unenhanced axial CT images of a 57-year-old male patient with non-severe COVID-19 pneumonia. (**a**) Chest CT showed the ill-defined ground-glass opacity in the middle lobe of the right lung. The lung severity score was 0.4. (**b**) No abnormal findings were observed in the pancreas and liver on the abdominal CT. The CT-FVF of the pancreas was 4.5 % and the CT attenuation value of the liver was 61.2 HU.

The automated segmentation of the pancreas using unenhanced CT was successfully conducted without manual modification in all patients. The median values of CT-FVF (%) of the pancreas were 11.0% (interquartile range [IQR], 15.4), and the median lung severity score was 1.0 (IQR, 2.0). The CT-FVF (%) values of the pancreas had a significantly positive correlation with the lung severity score on CT (*ρ* = 0.549, *p* < 0.01) (Figure 3 and Figure 4). The median CT attenuation value (HU) of the liver on unenhanced CT was 58.0 (IQR, 14.1). There was a significant correlation between the CT attenuation value (HU) of the liver and the lung severity score on CT (*ρ* = −0.376, *p* < 0.01). The age was significantly correlated with the CT-FVF (%) values of the pancreas (*ρ* = 0.421, *p* < 0.01) and the lung severity score (*ρ* = 0.450, *p* < 0.01), but not with the CT attenuation value (HU) of the liver (*ρ* = −0.013, *p* = 0.88).

Among the 128 COVID-19 patients, 33 had severe pneumonia, while 95 had non-severe pneumonia. The results of the comparison of the CT-FVF (%) of the pancreas as well as CT attenuation value (HU) of the liver between the severe and non-severe pneumonia groups are shown in Table 1. The CT-FVF (%) of the pancreas in the severe pneumonia group (21.7%, IQR 15.5) was significantly higher than that of the non-severe pneumonia group (7.8%, IQR 11.8) (*p* < 0.01). The median CT attenuation value (HU) of the liver in the severe pneumonia group (53.4 HU, IQR 14.2) was significantly lower than that of the non-severe pneumonia group (59.9 HU, IQR 12.9) (*p* = 0.01).

**Figure 5:**
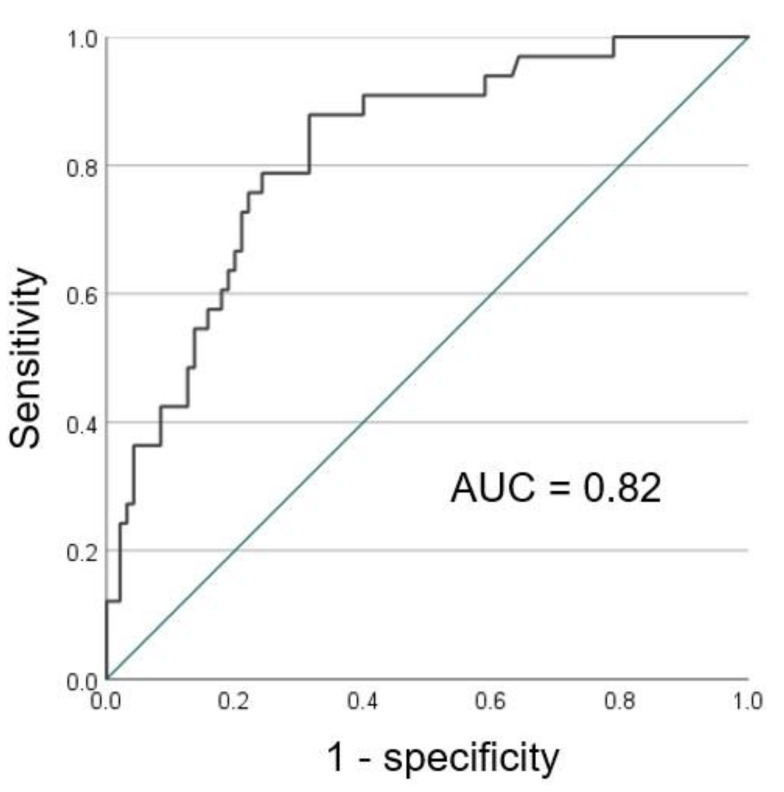
The ROC curve of CT-FVF (%) of the pancreas predicting the severity of pneumonia on CT.

The AUC of CT-FVF (%) of the pancreas in predicting the severity of pneumonia in CT was calculated to be 0.82, with a sensitivity of 88% and a specificity of 68% at a threshold of 12.3 (Figure 5). Conversely, the AUC of CT attenuation values (HU) of the liver in predicting the severity of pneumonia in CT was rendered to be 0.65, providing a sensitivity of 76% and a specificity of 53% at a threshold of 59.4.

## 4. Discussion

This study showed that the CT-FVF (%) of the pancreas calculated using a histogram analysis with threshold CT attenuation values of −20 HU after the automated segmentation and volumetry of the pancreas on unenhanced CT had a significantly positive correlation with the CT lung severity score in patients with COVID-19 pneumonia. In addition, the CT-FVF (%) of the pancreas had a better AUC value with a higher sensitivity and specificity in predicting the severity of pneumonia than the CT attenuation value (HU) of the liver. This result indicated that the automated volumetric measurement of the pancreatic CT-FVF (%) was more suitable for estimating the severity of COVID-19 pneumonia than measurements of CT attenuation of the liver.

Obesity, diabetes and other metabolic risk factors have been shown to be strongly associated with an increased severity of COVID-19 disease [15,16,17]. Under conditions of obesity, there is chronic low-level systemic inflammation characterized by increased secretion of adipokines which are inflammatory cytokines from adipose tissue. Furthermore, the COVID-19 virus is known to bind angiotensin-converting enzyme 2 (ACE2), and while ACE2 in lung tissue is considered an important site of COVID-19 virus entry, ACE2 expression in adipose tissue is even higher than in the lungs, which is thought to increase vulnerability to COVID-19 virus in obese individuals [18,19]. Based on these factors, adipose tissue, including organ adipose tissue, might be capable of promoting acute disease through increased inflammation at the visceral organs [18].

Similar to hepatic steatosis, pancreatic steatosis is regarded as a pancreatic manifestation of metabolic syndrome, including insulin resistance, diabetes, and obesity. Therefore, pancreatic steatosis as an indicator of the metabolic syndrome can be considered a predisposing factor for severe COVID-19. Furthermore, compared with the evaluation of hepatic steatosis on chest CT, pancreatic steatosis might be more efficiently evaluated, since the pancreas is mostly covered by chest CT, while the liver is not entirely visualized.

Pancreatic steatosis has been evaluated on unenhanced CT using ROI measurements, including the correlation with COVID-19 infection [11,20]. However, fat distribution in the pancreas is often inhomogeneous [21,22], and thus, the areas of the measured ROIs may not represent the fat deposition of the entire pancreas. The CT-FVF (%) of the pancreas, calculated from the automated volumetric measurements, may lead to the acquisition of more accurate pancreatic fat volumes on CT than with the ROI measurements obtained randomly on selected slices. This suggests that the automated volumetric measurement of the pancreatic CT-FVF (%) is more suitable for quantifying pancreatic steatosis than ROI measurements of the pancreatic CT attenuation.

Several limitations associated with the present study warrant mention. First, patients with typical CT findings of COVID-19 pneumonia but with a negative PCR test were not included in this study, which may have led to the loss of potential COVID-19 pneumonia patients. Second, the pancreatic fat volume was not confirmed histologically because of the technical difficulty of a pancreatic biopsy and ethical issues. However, this study aimed to investigate the relationship between the severity of pneumonia based on chest CT findings and that of pancreatic steatosis estimated by CT-FVF (%). Third, CT scans of all patients could not be performed on the same symptomatic day because they presented on different days after the onset of their symptoms. Fourth, other diseases in patients other than COVID-19 were not evaluated because of the retrospective nature of this study. Further prospective studies are needed to validate our results. Fifth, clinical outcomes were not compared in this study, since most patients received general hospitalization and were not treated in the intensive-care unit, and all were discharged with full recovery. If an association between pancreatic steatosis and worse clinical outcomes in patients with COVID-19 is confirmed by further studies, then the role of chest-abdominal CT may be expanded to include the estimation of the severity of pneumonia and the prediction of the prognosis. Finally, these results were obtained during a limited period of the pandemic. Therefore, the clinical role of pancreatic steatosis in patients with COVID-19 must be verified during study periods with different disease severities.

## 5. Conclusions

The automated volumetric measurement of the CT fat fraction of the pancreas using unenhanced CT will aid in estimating disease severity in patients with COVID-19 pneumonia based on chest CT findings. Assessing the risk of severity of COVID-19 not only based on pneumonia but also pancreatic steatosis may offer an important insight into the pathogenesis of the infection.

## Figures and Tables

**Table 1:** Comparison of CT-FVF (%) of the pancreas and CT attenuation value (HU) of the liver between the severe and non-severe pneumonia groups.

	Severe Pneumonia Group (*n* = 33; Male = 24, Female = 9)	Non-Severe Pneumonia Group (*n* = 95; Male = 56, Female = 39)	*p*-Value
CT-FVF (%) of the pancreas	21.7 (15.5)	7.8 (11.8)	<0.01
Male	21.4 (12.0)	9.4 (9.1)	<0.01
Female	24.4 (49.3)	5.7 (12.3)	<0.01
CT attenuation value (HU) of the liver	53.4 (14.2)	59.9 (12.9)	0.01
Male	51.1 (19.1)	54.9 (23.2)	0.09
Female	57.6 (7.8)	61.2 (9.2)	0.16

Data are medians, with interquartile range in parentheses.

## Data Availability

Not applicable.

## Abbreviations

ACE2	angiotensin-converting enzyme 2
AUC	area under the curve
COVID-19	coronavirus disease 2019
DM	diabetes mellitus
FVF	fat volume fraction
IQR	interquartile range
PACS	picture archiving and communication system
PCR	polymerase chain reaction
ROI	region of interest
3D	three-dimensional
